# Association between gestational weight gain and metabolic and inflammatory biomarkers in the ETCHED cohort

**DOI:** 10.1038/s41598-026-41560-w

**Published:** 2026-02-25

**Authors:** Sourav RoyChoudhury, Rachel Caballero, Dorota Wasak, Yunhua L. Muller, Zaqueena Coleman, William C. Knowler, Robert L. Hanson, Marilyn Q. Sale, Madhumita Sinha

**Affiliations:** Diabetes Epidemiology and Clinical Research Section, National Institute of Diabetes and Digestive and Kidney Diseases (NIDDK), Phoenix Epidemiology and Clinical Research Branch (PECRB), National Institutes of Health (NIH), 850 N 5th St. Suite 506, Phoenix, AZ 85004 USA

**Keywords:** Pregnancy, Gestational weight gain, Biomarkers, Adverse pregnancy outcome, Biomarkers, Diseases, Endocrinology, Medical research, Risk factors

## Abstract

Gestational weight gain (GWG) is a critical determinant of maternal and neonatal health, with both excess (EGWG) and inadequate GWG (IGWG) linked to adverse pregnancy outcomes and metabolic risk. We investigated the association between maternal prenatal demographic, clinical, and metabolic biomarkers at baseline, and GWG in singleton pregnancies from the Early Tracking of Childhood Health Determinants (ETCHED) life-course cohort. GWG, defined as the difference between pregestational and pre-delivery weight, was the primary outcome. Among 120 eligible mothers, 79.2% self-identified as Hispanic and 16.0% as American Indian/Alaska Native. The cohort represented socioeconomically disadvantaged populations: 75% had a high school education or less, 31% were single mothers, and 52.5% reported an annual household income < $25,000. Compared with mothers with normal GWG, those with EGWG had significantly higher leptin and lower IL-8 levels (*P* < 0.05). A multivariate multinomial logistic regression identified leptin to be strongly associated with EGWG (OR/1 SD: 11.09; 95%CI 3.18–38.67; *P* = 0.001), followed by FGF21, IL8, and pregestational BMI. Higher cortisol levels were associated with increased risk of IGWG (OR/1 SD: 2.59; 95%CI 1.07–6.29; *P* = 0.03). EGWG was associated with higher rates of cesarean delivery and prematurity. These findings highlight distinct metabolic and inflammatory biomarkers associated with GWG in an understudied population.

**Supplementary Information:**

The online version contains supplementary material available at 10.1038/s41598-026-41560-w.

## Introduction

The prevalence of pregestational obesity among reproductive age women across the United States (US) has shown a steady rise in recent years with almost 3 in 10 women suffering from obesity prior to conception^1^. A similar trend has also been observed with gestational weight gain (GWG) that exceeds the specified limits recommended in the 2009 National Academy of Medicine (NAM, previously the Institute of Medicine) guidelines^2–5^. Currently it is estimated that approximately half of pregnant women in the US achieve excess GWG with a higher proportion of such gain occurring in those with pre-existing overweight or obesity^3^. Although maternal weight gain in pregnancy is a normal physiological process and is essential for sustaining optimal fetal growth and development, excess gain is primarily associated with accrual of maternal fat mass^6^. Pregnancy is also a period when maternal immunologic adaptation occurs to a semi allogenic fetus, hence systemic inflammatory conditions such as pregestational obesity and excess GWG may exaggerate maternal immune activation and increase the risk of metabolic dysfunction^7,8^. Higher insulin resistance leads to hyperglycemia, and dyslipidemia, resulting in impaired glucose tolerance, gestational diabetes, and preeclampsia^9,10^. Just as excess weight gain is detrimental to maternal–fetal health, inadequate pregnancy weight gain, even in women with overweight and obesity, also poses a risk for adverse health outcomes including preterm labor, decreased neonatal fat and lean body mass, and birth of a small for gestational age neonate (SGA)^11,12^.

In a recent systematic review and meta-analysis of over a million pregnancies, gestational weight gain at both extremes, inadequate or excess, was associated with adverse pregnancy outcomes (APO)^13^. Those mothers who gained weight below the NAM guidelines showed a higher risk for birth of preterm and SGA neonates, whereas those with excess GWG had a higher risk for large for gestational age (LGA) neonates, fetal macrosomia, and cesarean section deliveries^13^. Additional studies have supported similar observations that pregestational body mass index (BMI) has a robust association with APOs, however the definition of optimal gestational weight gain remains elusive, and its relationship with pregnancy outcomes varies largely based on population characteristics, outcomes examined, analytical methods, and individual study specific factors^14–16^.

Although recent literature suggests that APOs may occur across the pregestational BMI range and at both ends of the weight gain spectrum, few studies have explored the metabolic alterations that are associated with GWG. Here we hypothesize that maternal prenatal metabolic profile will differ based on NAM recommended GWG categories of inadequate, normal, and excess weight gain. The objective of the current study is to characterize maternal prenatal serum metabolic and inflammatory profile across the GWG spectrum in a high-risk pregnancy cohort and identify specific biomarkers that are associated with excess or inadequate maternal weight gain.

## Methods

### Study participants

Clinical and biomarker data were obtained from the ongoing Early Tracking of Childhood Health Determinants (ETCHED) study. ETCHED is a longitudinal observational life course study of Hispanic and American Indian/Alaska Native (AI/AN) pregnant women and their offspring, that examines the biological and environmental factors contributing towards childhood obesity and metabolic risk^17^. Pregnant women ≥ 18 years were enrolled from the antenatal clinics at Valleywise Health hospital, in Maricopa County, Arizona. Women with a viable pregnancy are enrolled any time during the prenatal period until delivery; the first research examination post-enrollment was considered as the “baseline examination”. Data from prenatal maternal baseline research visits and delivery events were analyzed. Among the 128 participants who completed their baseline exam and had data available for analysis, three had multiple fetuses, and five did not have pregestational weight recorded and were excluded. A final cohort of 120 women and their offspring were included.

### Measures

*Maternal characteristics*: Demographic and clinical information obtained at baseline examination included: maternal age, self-reported race/ethnicity, gravida, and gestational age (in weeks), history of diabetes, and gestational hypertension and/or preeclampsia. Maternal weight (in kilograms) and height (in centimeters) were recorded at the baseline visit. In addition, blood was drawn (fasting) for clinical testing and biomarker measurements. At delivery, maternal weight, delivery mode, gestational age (in weeks), pregnancy and delivery related complications, were obtained from patient’s electronic health records (EHR).

GWG was the outcome of interest and was based on the difference between the last recorded pre-delivery weight and pregestational weight. A comprehensive review of the patient’s EHR was performed for any clinical visits where a measured weight was documented (non-pregnant) during the 12-months preceding the last menstrual period. If unavailable, then the first weight from the prenatal record (≤ 16-weeks’ gestation) in the current pregnancy was taken. In the absence of either, a maternal self-reported pregestational estimated weight was accepted, and measured height at the baseline visit were used to calculate the pregestational BMI. Maternal pregestational weight category was determined by the CDC’s published criteria for adults^18^. Further, patients were assigned to three categories of weight gain during pregnancy as recommended by the NAM guidelines: excess GWG, normal GWG, and inadequate GWG (IGWG)^19^. The recommended GWG for women with pregestational BMI in the underweight range (< 18.5 kg/m^2^) is 12.5–18 kg, normal (BMI:18.5- < 25.0 kg/m^2^) is 11.5–16 kg, overweight (BMI:25- < 30.0 kg/m^2^) is 7–11.5 kg, and obesity (BMI: ≥ 30 kg/m^2^) is 5–9 kg^19^. Pregnant women were assigned to the different weight gain categories based on their individual pregestational BMI and recommended weight gain target.

*Offspring characteristics:* obtained at delivery from EHR included neonatal sex, birthweight and length, congenital anomalies, length of hospitalization, disposition, and neonatal complications.

*Biomarker selection and measurements from maternal prenatal serum*: Fourteen metabolic and inflammatory biomarkers were selected based on review of published literature related to maternal obesity, GWG, and changes in inflammatory biomarkers. Maternal obesity has been associated with a low grade chronic inflammatory state that is further altered with pregnancy and excess weight gain, and may lead to complications such as GDM^20–22^. A recent study by Saucedo et al. showed that gene expression in visceral adipose tissue associated with inflammatory pathways such as IL-6 and TNF-α, was higher in pregnant women with excess and adequate GWG compared to those with inadequate GWG. In addition to examining adipocytokines leptin and adiponectin^23,24^, and proinflammatory biomarkers TNF-α and interleukins, based on their importance in GWG, we also examined additional metabolic markers retinol binding protein 4 (RBP4) and fibroblast growth factor 21 (FGF21)^25^, that have association with maternal obesity and GWG.

The serum adipocytokines, leptin and adiponectin, proinflammatory markers IL-6, IL-8, TNF-α, and metabolic regulator proteins including RBP4 and FGF21, and apolipoprotein C3 (APOC3) were measured using enzyme-linked immunosorbent assay (ELISA). All specimens were measured and analysed in duplicates, and the standard curves for the proteins were created using four parameters logistic fit except for leptin and adiponectin, which were calculated using linear regression. C-reactive protein (CRP) was measured using a nephelometric assay. Fructosamine, a glycated serum protein reflecting short-term glycemia, was measured by employing a two-step enzymatic assay on Siemens Dimension EXL 200 integrated chemistry system. Insulin, C-peptide, and cortisol were measured using a two-site immunoenzymometric assay and a competitive enzyme immunoassay respectively. Glycated hemoglobin (HbA1c) was measured in venous blood using a high-performance liquid chromatography. All biomarker measurements were performed from blood samples collected at baseline exam following overnight fasting, within 3-months and onsite at the National Institute of Diabetes and Digestive and Kidney Disease’s (NIDDK’s) laboratory in Phoenix, AZ.

### Statistical analyses

Continuous variables are presented as mean and standard deviation or median and interquartile range. For categorical variables, number and percentage are reported. Spearman’s partial correlation coefficient was used to investigate pairwise associations between biomarkers from maternal prenatal blood samples with covariates adjustment for, pregestational BMI, maternal age (years), and gestational age (weeks) at blood draw, and diabetes in pregnancy. Medians of individual biomarker levels in the three GWG categories, inadequate, normal, and excess, were compared for statistical significance using the non-parametric Kruskal–Wallis test.

The multivariate imputation by chained equations (MICE) algorithm was used to impute missing values^26^. For comparing degrees of biomarker association measured using different units and concentration ranges, standardization methods (ORs scaled to 1 SD increments) were deployed.

From the initial set of 20 variables (relevant clinical/metabolic/inflammatory biomarkers) univariate analysis was performed, 18 variables that showing significance in the univariate analysis (*P* < 0.01), were included in the multinomial logistic regression analysis. The multinomial logistic regression model was selected to explore the effect the biomarkers and clinical parameters on the multiclass outcome variable, i.e. GWG classified into: EGWG, normal GWG, and IGWG^2^. The clinical variables included were pregestational BMI, maternal age, and gestational age at prenatal baseline visit, history of diabetes in pregnancy, and preeclampsia and/or gestational hypertension. The clinical variables, Cohen’s perceived stress scale 4 (PSS-4), and adverse childhood experiences (ACE) were not significant in the univariate analysis and were not included in the multinomial logistic regression model. Receiver operating characteristic (ROC) curves were generated to evaluate multinomial logistic regression model performance predicting EGWG and IGWG from normal GWG. All statistical analyses were performed using R Statistical Software (v4.4.2; R Core Team 2024).

All research was approved by the Institutional Review Board of the National Institutes of Health (NIH IRB # 18-DK-N071; NCT03481829; https://clinicaltrials.gov/study/NCT03481829?cond=ETCHED&rank=1). The NIH IRB approved protocols are compliant with the U.S. Department of Health and Human Services Federal Policy for the Protection of Human Subjects (the Common Rule 2018; https://www.hhs.gov/ohrp/ regulations-and-policy/regulations/45-cfr-46/revised-common-rule-regulatory-text/index.html). NIH IRB approved studies are also required to abide by the Declaration of Helsinki, and Belmont Report (https://www.hhs.gov/ohrp/regulations-and-policy/belmont-report/ index.html) that outline the ethical principles and guideline for the protection of human subjects participating in research. All participants underwent detailed informed consent procedure prior to participation and signed consents that were available in both English and Spanish.

## Results

### Cohort description (maternal and neonate)

Among 120 pregnant women enrolled between May 2022 and February 2025, median gestational age at baseline exam was 33 weeks (26.0–36.0) and were comparable between EGWG, normal GWG, and IGWG groups (*P* > 0.05, Table 1). The ETCHED study allows pregnant women who fulfill eligibility criteria to enroll in the study at any time during pregnancy, 26% of baseline exams were conducted in the second, and 72% in the third trimester of pregnancy in this cohort. Fifty-three women were assigned to the EGWG group, 38 to the normal GWG, and 29 participants were in the IGWG group. Median maternal age was 29.0 years (24.0–34.8). 79.2% were self-reported Hispanic ethnicity with 16% were AI/AN race. There was a significant within group difference in self-reported Hispanic ethnicity, however no significant self-reported race differences were observed (Table 1). Most mothers in the cohort were from socioeconomically disadvantaged backgrounds: 75% had a high school education or less, 31% were single mothers, and 52.5% reported an annual household income below $25,000. However, there were no significant differences in maternal education, current living situation, annual household income, or family size across the three GWG groups (Table 1). Only one participant reported current or prior smoking, and this did not influence any outcomes or observations in the study cohort. Median pregestational BMI was 31.7 kg/m^2^ (26.1–36.4), and median GWG was 10.2 kg (6.7–14.4). Thirty-two (26.7%) women had diabetes in pregnancy, of them, 10 had pregestational type 2 diabetes., and 14.2% had gestational hypertension and/or preeclampsia. Women with EGWG had a higher proportion of cesarean section (47.1%) and preterm deliveries (15.7%) compared to normal and IGWG mothers (Table 1).

**Table 1:** Maternal and neonatal demographics and clinical characteristics

Characteristics	Excess GWG^#^ (n = 53)	Normal GWG (n = 38)	Inadequate GWG (n = 29)	*P* value
Maternal age (Median, IQR)	27 (24–36)	28.5 (24–33)	30 (27–35)	0.53
Hispanic (n, %)	37 (69.81)	32 (84.2)	26 (89.65)	0.02*
Race (n, %)				
White American Indian/Alaska Native (AI/AN) Unknown/not reported Multiple race/other	9 (16.98) 12 (22.64) 29 (73.58) 3 (5.66)	9 (23.7) 4 (10.5) 24 (63.2) 1 (2.6)	6 (20.69) 3 (10.34) 20 (68.97) 0	0.42 0.07 0.19 0.16
Maternal education (n, %)				0.8
College degree or attended 1–3 years of college, business, or technical school High School, completed and/or in part Less than High School Not reported	11 (20.8%) 34 (64.2%) 6 (11.3%) 2 (3.8%)	6 (15.8%) 27 (71.1%) 4 (10.5%) 1 (2.6%)	8 (27.6%) 15 (51.7%) 4 (13.8%) 2 (6.9%)	
Mother’s current living situation (n, %)				0.94
Married Unmarried Unmarried but living with a partner Separated/Divorced Widowed Other/not reported	12 (22.6%) 13 (24.5%) 23 (43.4%) 1 (1.9%) 1 (1.9%) 3 (5.7%)	9 (23.7%) 12 (31.6%) 14 (36.8%) 2 (5.3%) 0 1 (2.6%)	9 (31%) 6 (20.7%) 11 (37.9%) 2 (6.9%) 0 1 (3.4%)	
Annual household income (self-reported) (n, %)				0.89
< $25,000 $25,000–< $50,000 $50,000–< $75,000 ≥ $75,000 Not reported	27 (50.9%) 14 (26.4%) 7 (13.2%) 1 (1.9%) 4 (7.5%)	20 (52.6%) 12 (31.6%) 2 (5.3%) 0 4 (10.5%)	16 (55.2%) 6 (20.7%) 3 (10.3%) 1 (3.4%) 3 (10.3%)	
Family size (Median, IQR)	4 (3–5)	5 (3.25- 6)	4 (3–5.25)	0.42
Current/history of smoking	1 (1.9%)	0	0	0.53
Gestational age in weeks at baseline visit (Median, IQR)	33 (27–35)	33 (28–36)	32 (24–36)	0.69
Pregestational Body Mass Index (Kg/m^2^) (Median, IQR)	32.9 (28–36)	28.75 (25.0–38.6)	32.10 (23.7–36.0)	0.31
Gravida (Median, IQR)	3 (2–5)	3 (2–4)	4 (2–5)	0.20
Diabetes in pregnancy (n, %)	13 (24.53)	10 (26.3)	9 (31.03)	0.52
Hypertensive Disorders of Pregnancy (n, %)	10 (18.87)	4 (10.5)	3 (10.34)	0.15
Perceived stress scale 4 (Median, IQR)	6 (3–8)	6 (3.75–8)	4.5 (2–6.75)	0.20
Adverse childhood experiences (Median, IQR)	1.5 (0–4)	1 (0–3)	2 (0–3)	0.76

Pregnancy outcomes, maternal and neonatal^$^	(n = 51)	(n = 37)	(n = 29)	*P* value
Gestational age in weeks at delivery (Median, IQR)	39 (38–39)	39 (38–39)	39 (37–39)	0.94
Delivery mode (n, %)				
Cesarean section Normal vaginal delivery	24 (47.06) 27 (52.94)	11 (29.7) 26 (70.3)	6 (20.69) 23 (79.31)	0.01*
Neonatal sex (n, %)				
Male Female	29 (56.86) 22 (43.14)	15 (40.5) 22 (59.5)	12 (41.38) 18 (62.07)	0.09
Prematurity (< 37 weeks) at birth (n, %)	8 (15.69)	1 (2.7)	2 (6.9)	0.03*
Birthweight in grams (Median, IQR)	3410 (2850–3880)	3310 (3010–3475)	3210 (2865–3450)	0.33
Congenital anomalies (major) (n, %)	2 (3.92)	3 (7.9)	1 (3.45)	0.31
Length of neonatal hospitalization (median, IQR)	2 (2–2)	2 (1–2.25)	1 (1–2)	0.049*

^#^Gestational weight gain; ^$^Includes information on in-hospital deliveries only; *P < 0.05 considered significant.

A total of 47.1% mothers with EGWG underwent cesarean-section deliveries that were significantly higher (*P* = 0.01) than in the normal GWG (29.7%) and IGWG groups (20.7%) (Table 1). Interestingly, the frequency of preterm delivery (≤ 37 weeks gestation) was significantly higher (*P* = 0.03) in mothers with EGWG (15.7%) compared to normal GWG (2.7%), and IGWG (6.9%). No significant association was observed between GWG and neonatal birthweight (r = 0.07, *P* = 0.43).

### Assessment of biomarkers at baseline prenatal exam

**Fig. 1:**
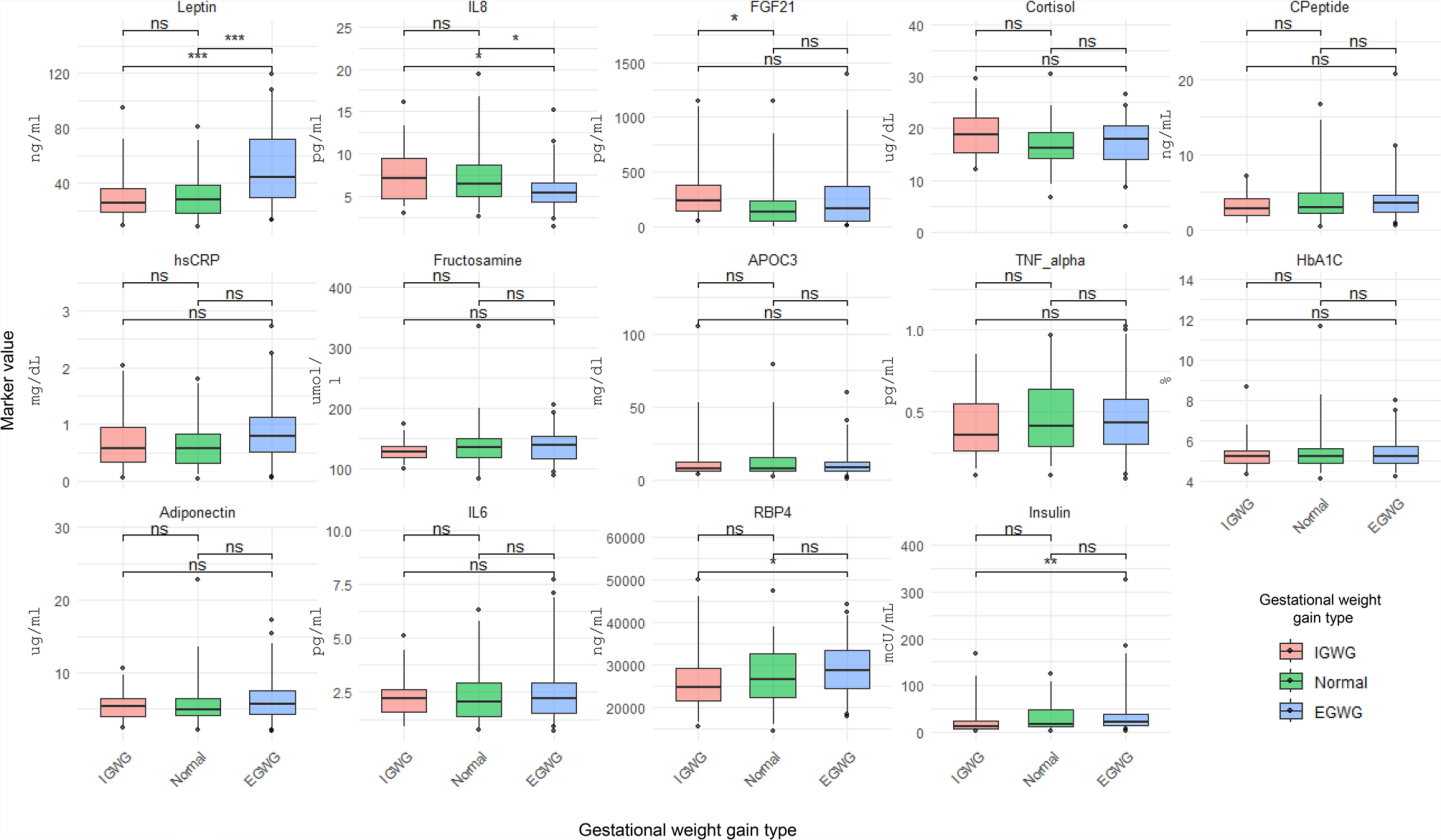
Box plots of baseline biomarker levels show the median, interquartile range, in different gestational weight gain categories. *P* values were derived from Mann–Whitney test; ***p ≤ 0.001, **p ≤ 0.01, *p ≤ 0.05.

Fourteen biomarkers (leptin, C-peptide, fructosamine, HbA1c, hsCRP, IL8, IL6, TNF-α, adiponectin, APOC3, cortisol, insulin, RBP4, and FGF21) were analyzed (Supplementary Table 1). Leptin levels at baseline prenatal exam were significantly higher in mothers with EGWG than in mothers with normal GWG, or IGWG (*P* < 0.001); no significant difference in leptin levels were observed between normal and IGWG groups (Fig. 1). In contrast, IL8 was significantly lower in EGWG than in normal GWG or IGWG (*P* < 0.05) pregnancies, and no difference was observed among normal GWG and IGWG. FGF21 levels were noted to be higher in mothers with IGWG than in normal GWG (*P* < 0.05). Both RBP4 (*P* < 0.05) and insulin (*P* < 0.01) levels were significantly higher in EGWG group than in mothers with IGWG (Fig. 1). These findings are further illustrated by quartile mapping of the biomarkers against mean GWG (Supplementary Fig. 1) and frequency distribution of GWG categories in each quartile (Supplementary Fig. 2).

### Correlations among serum biomarkers at baseline prenatal exam

**Fig. 2:**
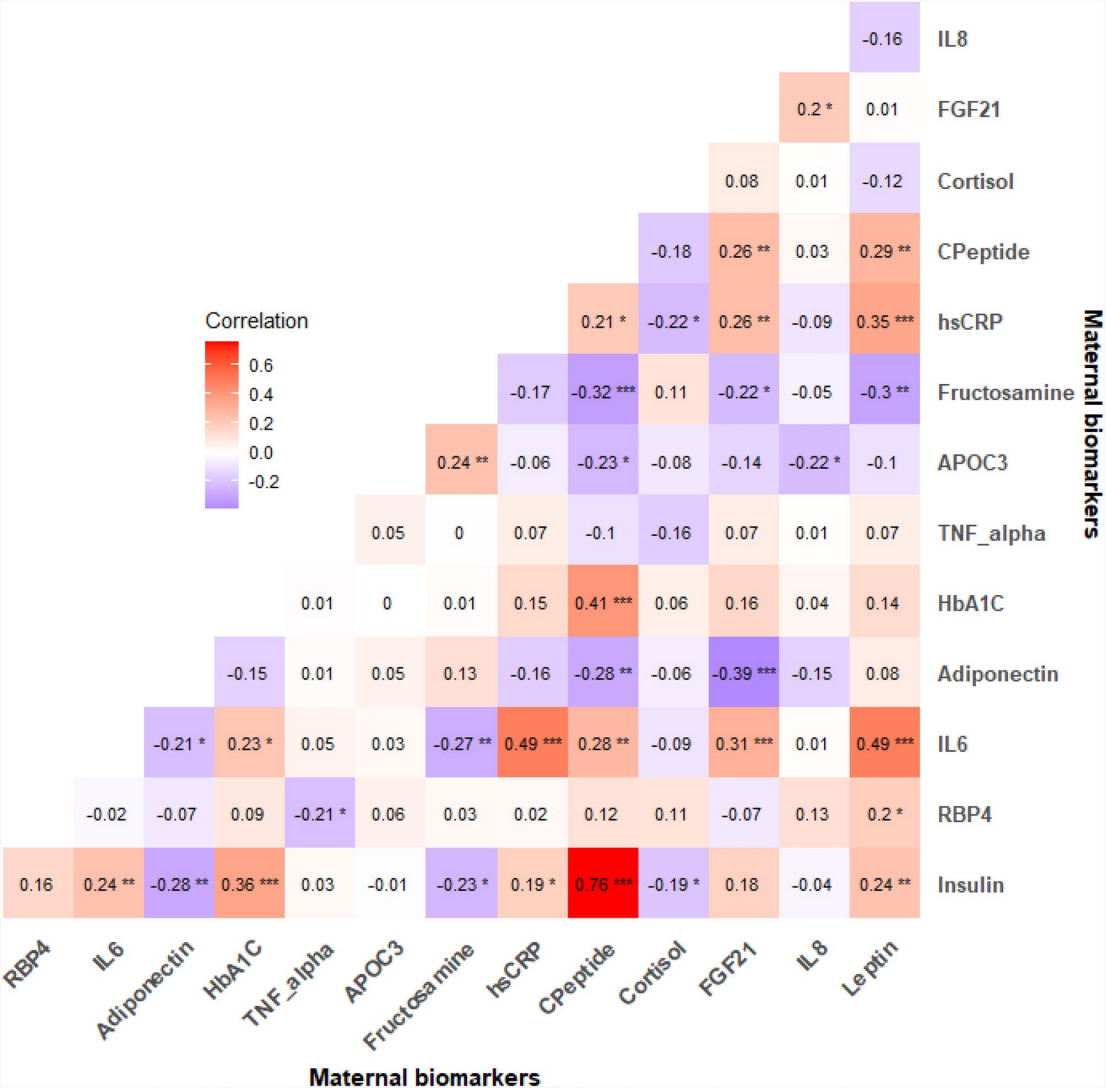
Spearman’s partial correlation matrix between maternal baseline biomarkers adjusted for gestational age, maternal age at baseline, and presence of diabetes. ***p ≤ 0.001, **p ≤ 0.01, *p ≤ 0.05.

Spearman’s partial correlation analysis between maternal prenatal metabolic and inflammatory biomarkers adjusted for gestational age at baseline, maternal age, and presence of diabetes, revealed important associations (Fig. 2). Leptin had a significant positive correlation (*P* < 0.05) with IL6, C-peptide, hs-CRP, insulin and RBP4. C-peptide was significantly positively correlated with insulin, HbA1c, IL6, and hs-CRP. FGF21 was positively correlated with IL6, C-peptide, hs-CRP; and negatively correlated with adiponectin. Higher levels of cortisol were significantly correlated with lower levels of insulin and hs-CRP. Additional correlations are shown in Fig. 2.

### Association of biomarkers with gestational weight gain

To explore the relationship and role of the biomarkers in GWG, a multinomial logistic regression model was applied. The outcome variable comprises three mutually exclusive categories of GWG: IGWG, normal GWG, and EGWG, with normal GWG as the reference group. The model included a total of 18 variables, 14 biomarkers (Supplementary Table 1), and 4 clinical variables. Leptin had the strongest association with EGWG (OR per 1 SD: 11.09; 95% CI 3.18–38.67; *P* < 0.001). Additionally, higher levels of FGF21 increased the risks of EGWG (OR per 1 SD: 3.15; 95% CI 1.37–7.25; *P* = 0.007). Conversely, higher levels of IL8 lowered the odds of EGWG (OR per 1 SD: 0.34; 95% CI 0.15–0.78; *P* = 0.01). Higher levels of C-peptide were associated with EGWG (OR per 1 SD: 0.36; 95% CI 0.14–0.96; *P* = 0.02) and IGWG (OR per 1 SD: 0.41; 95% CI 0.14–1.23; *P* = 0.11). In contrast, cortisol was associated with IGWG (OR per 1 SD: 2.59; 95% CI 1.07–6.29; *P* = 0.035). Among clinical variables, only maternal pregestational BMI was significantly associated with EGWG (OR per 1 SD: 0.82; 95% CI 0.72–0.93; *P* = 0.002).

**Fig. 3:**
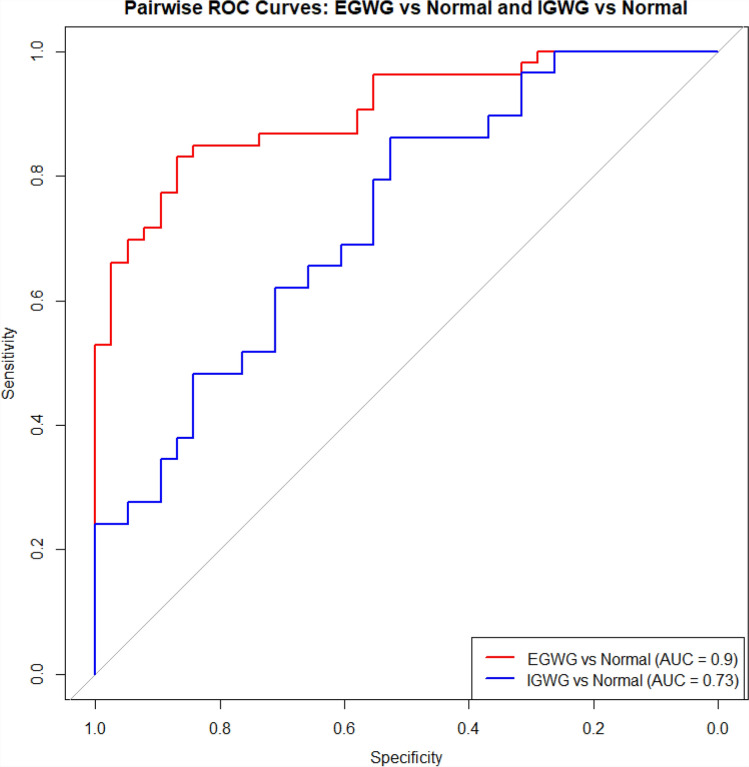
Receiver operating characteristic (ROC) curves evaluating the performance of a multivariable multinomial logistic regression model predicting EGWG (red line) and IGWG (blue line) from normal GWG.

The receiver operating characteristics (ROC) curves evaluating the multinomial logistic regression model performance predicting EGWG and IGWG from normal GWG showed good performance (Fig. 3). The area under the curve (AUC) predicting EGWG from normal GWG was 0.9, showing a strong accuracy for predicting EGWG. Similarly, AUC for predicting IGWG from normal GWG was 0.73 which indicates a moderate accuracy in predicting IGWG. Overall, the model was statistically significant and performed reasonably well, especially in predicting EGWG.

**Fig. 4:**
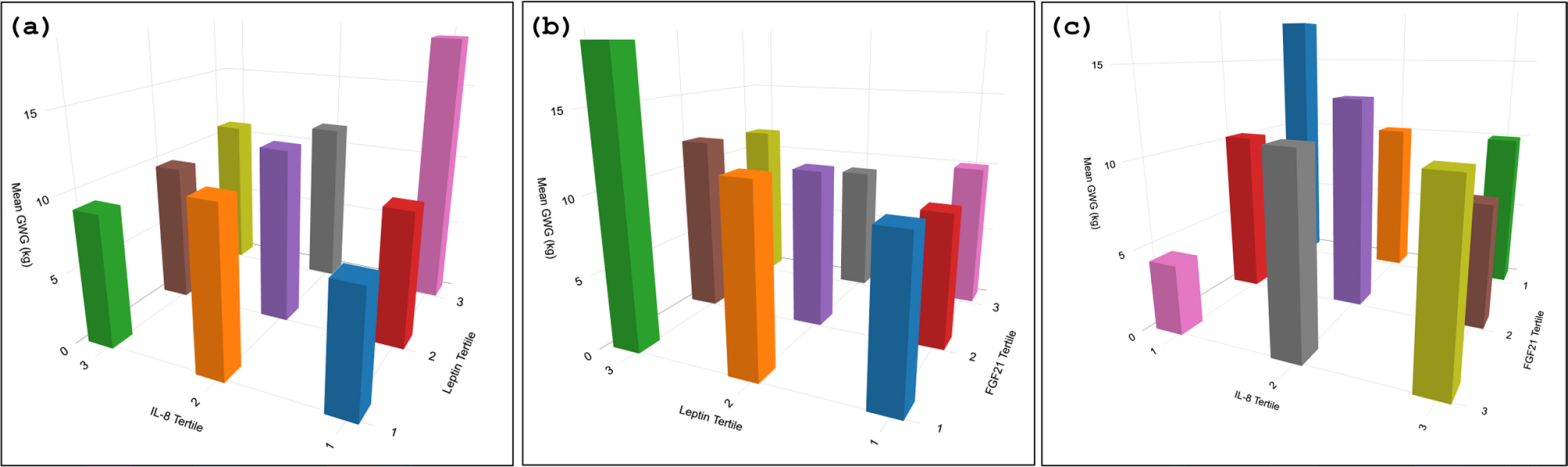
The panel shows mean GWG as a function of maternal biomarkers in tertiles where the combination of the variables, (**a**) Leptin and Il-8, (**b**) Leptin and FGF21, and (**c**) IL-8 and FGF21 are analyzed together. It shows the predicted GWG as a cross-classification of the two biomarkers.

To better delineate the effects of the significant biomarkers and their combined effects on GWG, the mean GWG was plotted as a function of prenatal serum biomarkers leptin, Il-8, and FGF21 (in tertiles). Figure 4 shows, mean GWG by a cross-classification of two biomarkers at a time. There was an interaction between leptin and IL-8 where the 1st tertile of IL-8 and the 3rd tertile of leptin levels (Fig. 4a) corresponds with a higher GWG. Similar bivariate relations between leptin and FGF21, and between IL8 and FGF21 for mean GWG are also presented in Fig. 4b and c, respectively. The greatest GWG was observed in those with the 3rd tertile of leptin and the 1st tertile of FGF21 (Fig. 4b); or in those with the 1st tertile of both IL8 and FGF21 (Fig. 4c). Leptin and IL8 have an almost linear relationship to mean GWG when plotted against their respective quartiles (Supplementary Fig. 1) and in the number of mothers with EGWG for each quartile of these biomarkers (Supplementary Fig. 2). However, the relations between FG21, C-peptide, and cortisol with mean GWG or the relative frequency of the mothers with EGWG were observed to be nonlinear in nature (Supplementary Figs. 1 and 2).

The frequency distribution plot of the 3 different GWG categories across the pregestational weight categories of healthy weight, overweight, and obesity classes I, II, and III, are shown in Supplementary Fig. 3. More than 50% occurrence of EGWG cases were in mothers with obesity classes I and II, and overweight categories, whereas IGWG was most frequently observed in pregestational healthy weight women.

## Discussion

In this cohort of Hispanic and AI women, we examined the metabolic and inflammatory biomarker profile during pregnancy and explored its association across the spectrum of gestational weight gain. With the rising trends in pregestational obesity among reproductive age women, a higher proportion of women now enter pregnancy with preexisting obesity and dysglycemia, that alters fetal growth and development and confers a higher risk of obesity and metabolic dysfunction in the offspring. This developmental programming triggered by adverse intrauterine exposures increases the offspring’s susceptibility to future chronic cardiometabolic diseases and forms the basis of the developmental origins of health and disease hypothesis described by Barker and his colleagues^27^, and validated by subsequent epidemiological studies^28,29^, These observations confirm previous studies among AIs^30,31^. They also explain the vicious cycle of transgenerational amplification of obesity and diabetes^30,31^. More than half of mothers entered pregnancy in our study with obesity, and over one-fourth had diabetes during pregnancy, higher than what has been reported nationally^1,32–34^. Also, over half the women with overweight and obesity, had excess gestational weight gain, and women with EGWG had a higher risk for cesarean section deliveries and preterm labor. Among the biomarkers, prenatal serum leptin was a significant predictor of excess GWG along with FGF21, and cortisol predicted inadequate weight gain. Leptin is an adipocyte derived peptide hormone, during pregnancy it is also produced by placental trophoblasts^35^. Leptin has a proinflammatory action and plays an important role in fetal growth and development^36^. Pregnant women with greater adiposity have elevated levels of circulating leptin and placental leptin resistance with altered leptin signaling^37,38^. Leptin was also significantly positively correlated with pregestational BMI, glycemic biomarkers C-peptide, Insulin, RBP4, and inflammatory biomarkers hs-CRP and IL-6 in this pregnancy cohort.

Prior studies have shown that plasma levels of FGF21 are elevated with insulin resistant states such as obesity, impaired glucose tolerance, and type 2 diabetes^39,40^. Similar associations between higher levels of FGF21 in pregnancy and an increased risk of gestational diabetes, particularly in those with overweight or obesity, have also been observed in recent studies^41–43^. In our study, the proportion of mothers with excess GWG is 44%, and inadequate GWG of 24% and these results consistent with large meta-analysis across diverse cohorts^13^. Serum cortisol that was primarily drawn in the fasting state during morning research visits in the pregnant mother was predictive of inadequate GWG.

The strength of the current study is the relatively large number of novel glycemic and inflammatory biomarkers examined in a high-risk underserved study population that has a high prevalence of obesity and diabetes^44,45^. Our study has limitations; it has an observational design that is susceptible to inherent confounding biases, however we have deployed appropriate methods in our analyses to mitigate this issue. Most of our participants enrolled in the study later in pregnancy therefore most did not have additional longitudinal examinations during the pregnancy, and this could impact the ability to adequately associate biomarker levels early in pregnancy with later trends in GWG. However, considering that our study participants suffer from health disparities and poor access to care, delayed prenatal care in minority women with poor social determinants of health is not unusual^46^. Also weight gain in normal pregnancies occurs primarily during the later trimesters of pregancy^47^, and the influence of gestational age at baseline exam was accounted for as a confounding factor in the statistical methods. Since the gestational ages at baseline exam had a wide range, the temporal relationships between the biomarkers and GWG are mixed, and the causal directions cannot easily be determined.

In summary, our study characterized maternal prenatal serum metabolic and inflammatory profile in an understudied population and identified specific biomarkers that are associated with pregnancy weight gain. However, the clinical utility from examining the novel biomarkers for abnormal pregnancy weight gain and its impact on future maternal-offspring metabolic health will require additional investigations that go beyond simple assessment of biomarker performance and the scope of the current study.

## Supplementary Information

Supplementary Information.

## Data Availability

The datasets generated and/or analyzed during the current study are not publicly available due to regulatory policies of the NIDDK, Phoenix Branch, that prohibit from sharing any individual-level clinical or research data publicly. However, data is available from the corresponding author on reasonable request that requires approval by the appropriate committees and the NIH IRB that approved this study.
