# Supplementary material for: Association between gestational weight gain and metabolic and inflammatory biomarkers in the ETCHED cohort
Source: Sci Rep. 2026 Feb 25;16:11066. doi: 10.1038/s41598-026-41560-w (PMC13044269; doi:10.1038/s41598-026-41560-w)
Supplement: Supplementary file 1 — Supplementary Information. [file 41598_2026_41560_MOESM1_ESM.pdf]

Association Between Gestational Weight Gain and Metabolic and Inflammatory Biomarkers in the ETCHED cohort

RoyChoudhury, S.; Caballero, R.; Wasak, D; Muller, Y. L.; Coleman, Z.; Knowler, W.C.; Hanson, R.L.; Sale, M.Q.; Sinha, M.

Supplementary figure 1

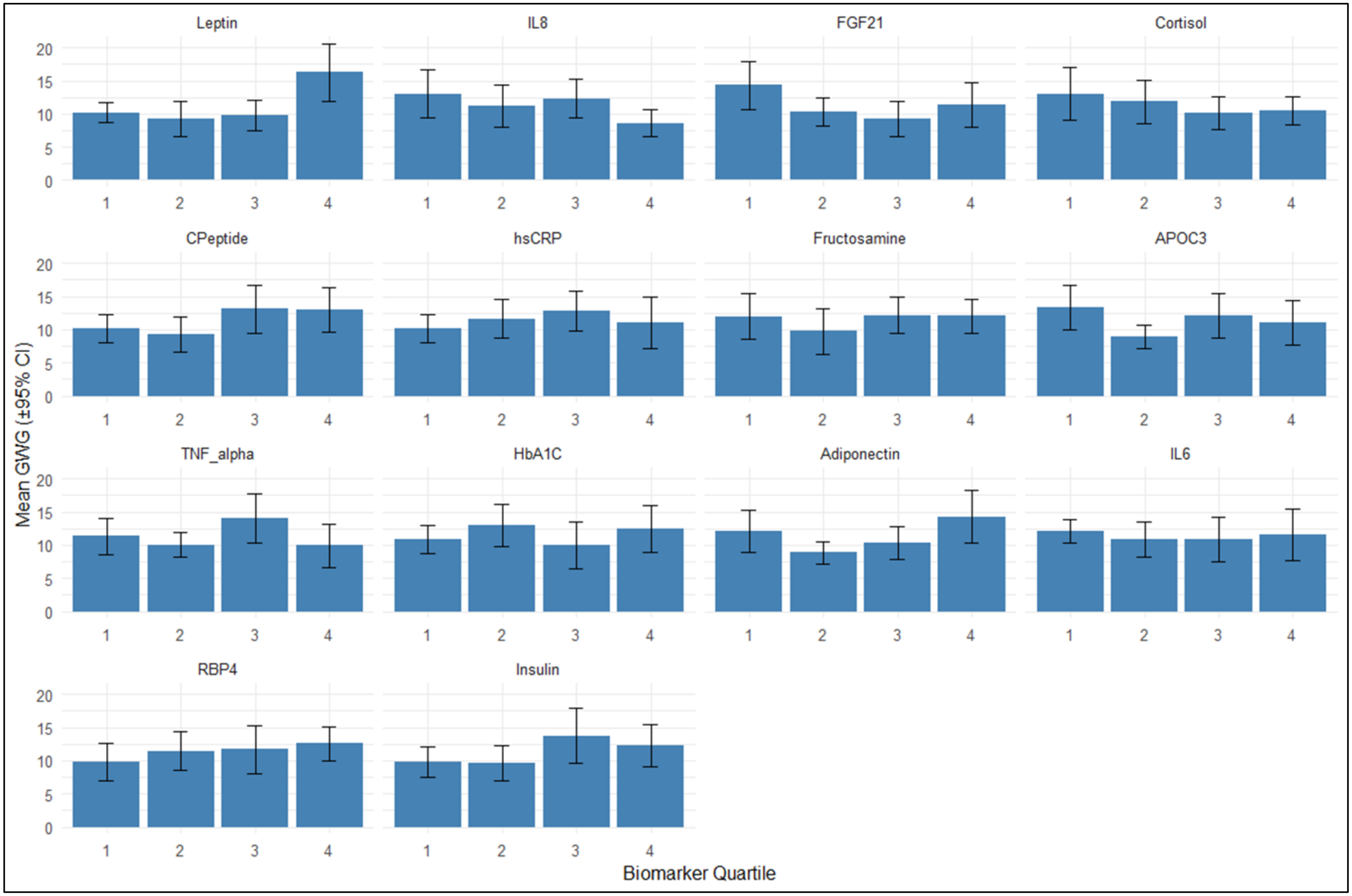

Gestational weight gain by biomarker quartiles shows the key predictor biomarker levels by their quartiles on the X axis and against mean GWG in kgs on the Y axis

Supplementary figure 2

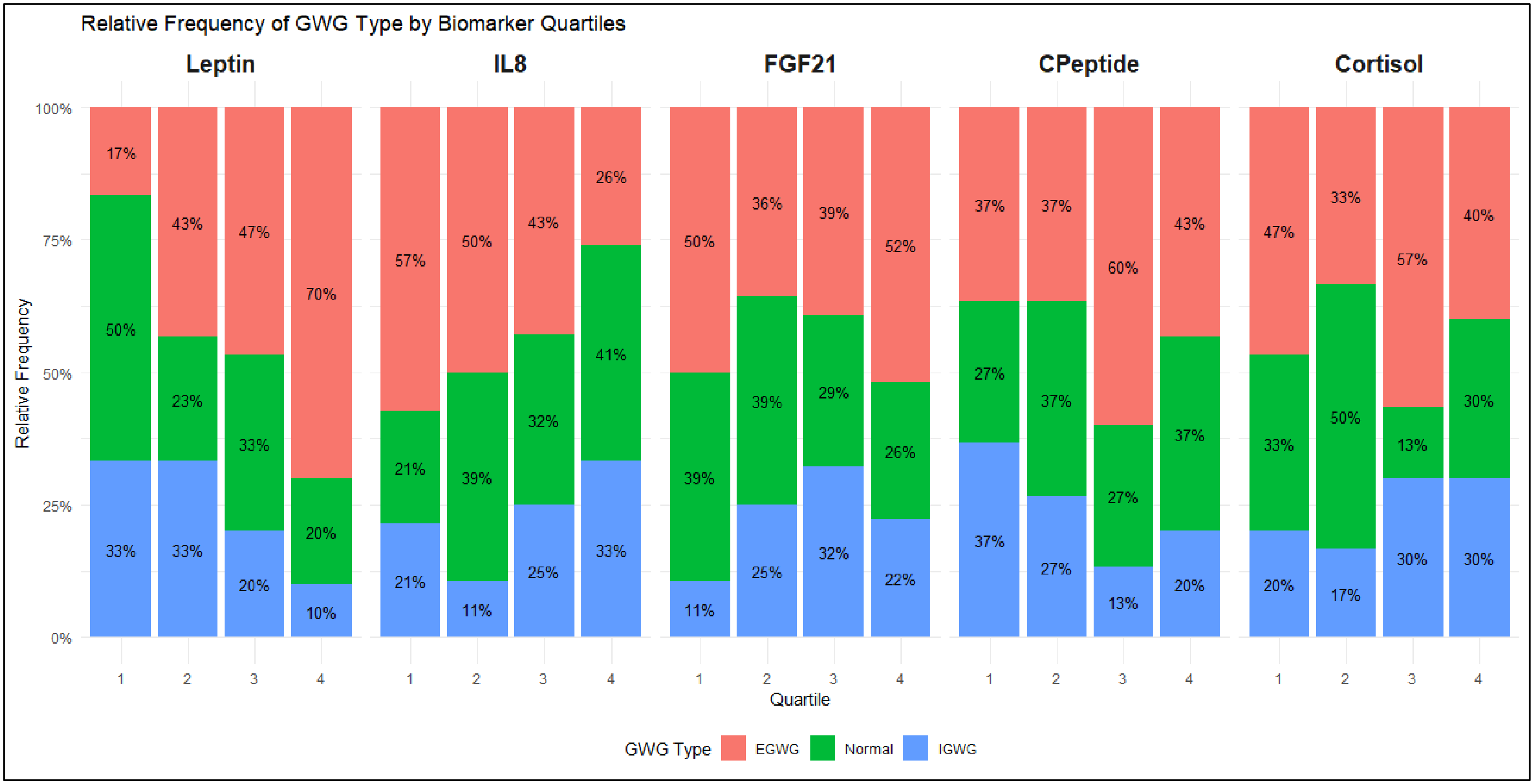

The key predictor biomarker levels by their quartiles on the X axis against relative frequency of the GWG categories on the Y axis

Supplementary figure 3

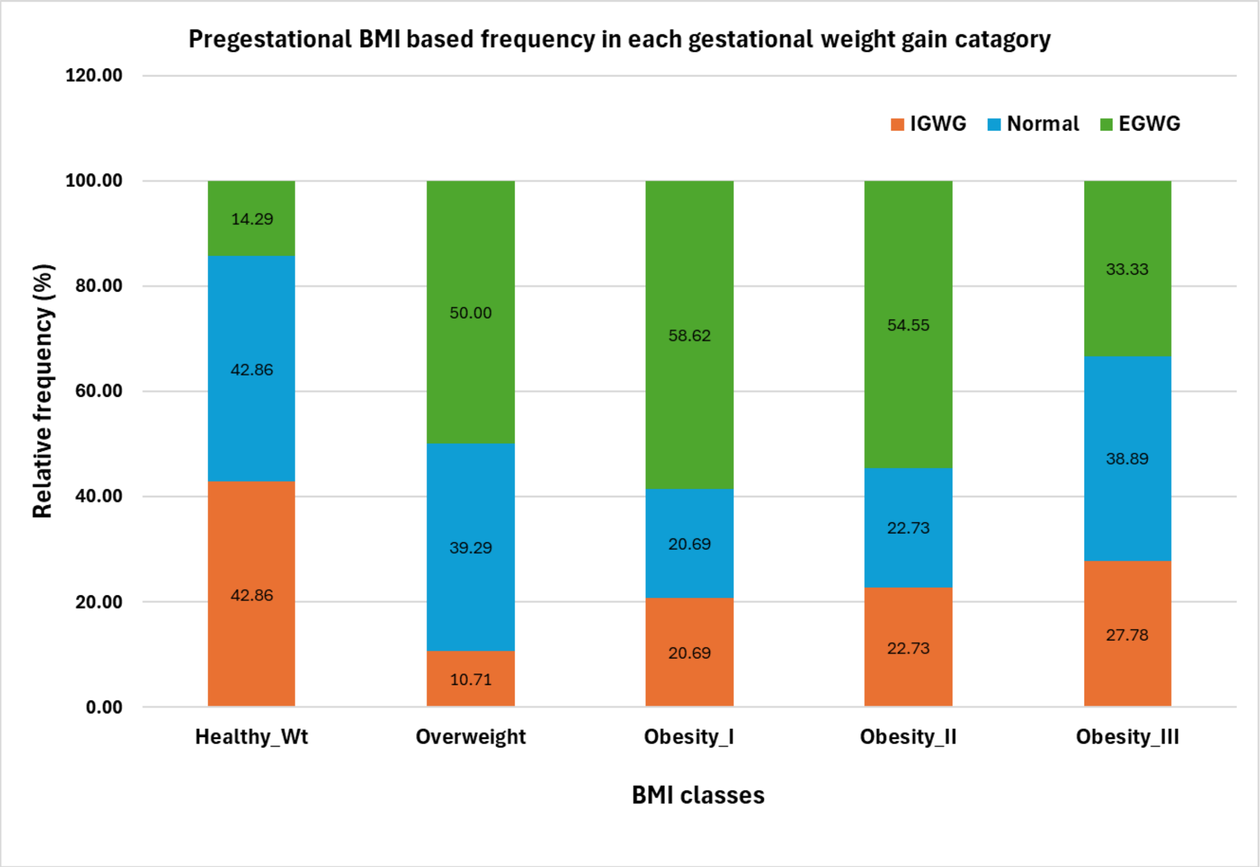

Relative frequency of GWG categories based on their pre gestational BMI shows differences in number of inadequate, normal and excess GWG cases in all the BMI categories. * 2 underweight participants, 1 in each of EGWG and IGWG group is not shown here

Supplementary table 1: Biomarker levels measured at maternal baseline prenatal examination in maternal blood/serum samples

Biomarkers	Minimum	Maximum	Mean	SD
C-Peptide (ng/mL)	0.50	20.75	4.02	3.18
Cortisol (ug/dL)	1.14	30.50	17.54	4.81
hs-CRP (mg/dL)	0.04	2.72	0.75	0.53
Fructosamine (umol/l)	83.00	334.00	136.06	30.14
Leptin (ng/ml)	8.62	119.57	40.07	25.35
Adiponectin (ug/ml)	1.89	22.84	5.86	3.03
TNF_alpha (pg/ml)	0.09	1.02	0.44	0.23
IL6 (pg/ml)	0.69	7.71	2.46	1.38
IL8 (pg/ml)	1.47	19.42	6.79	3.17
APOC3 (mg/dl)	0.91	104.90	12.75	14.31
RBP4 (ng/ml)	14,286.00	50,111.00	28,166.82	6,958.40
FGF21 (pg/ml)	0.00	1,395.59	246.59	274.09
HbA1C (%)	4.10	11.65	5.35	0.90
Insulin (mcU/mL)	1.10	327.10	33.82	43.61
